# Mechanisms of Covalent Bonds in Enhancing the Adsorption Stability of Clay–Polymer Gels in High-Temperature Environments

**DOI:** 10.3390/gels11080623

**Published:** 2025-08-09

**Authors:** Yu Wang, Fan Zhang, Liangbin Dou, Yutong Li, Kaiwen Wang, Zhengli Shi, Leyao Du, Wangyuan Zhang, Zonglun Wang

**Affiliations:** School of Petroleum Engineering, Xi’an Shiyou University, Xi’an 710065, China

**Keywords:** silicone polymer, covalent bond, gel system, high-temperature resistance, film-forming

## Abstract

To address the issue of drilling fluid performance drop and wellbore instability induced by desorption between treatment agents and clay in the high-temperature environment of ultra-deep drilling, this study synthesized three organosilicon polymers (ADE, ADM, ADD) with different substituents. The study confirmed that the covalent bond significantly improved the high-temperature adsorption resistance of clay, which is closely related to the interface behavior of gels. Through rolling recovery, rheology, and filtration experiments for performance evaluation, these organic silicon polymers showed excellent high-temperature performance: the shale rolling recovery rate exceeded 80% at 210 °C, and the filtration loss was reduced to 14 mL, with a reduction rate of 53.3%. The adsorption capacity of the three polymers on clay remained unchanged from 150 °C to 210 °C, among which the adsorption amount of trimethoxy groups stabilized at 8–11 mg/g after 150 °C. The adsorption capacity of ethoxy groups increased by 7.9% at 150–210 °C. The adsorption capacity of dimethoxy groups with methyl steric hindrance increased by 28.1% at 150–210 °C. These results indicate that covalent bonds effectively enhance the high-temperature adsorption of clay, allowing for polymer molecules to firmly anchor on the clay surface at high temperatures. This breakthrough overcomes the limitations of traditional inhibitors in high-temperature desorption, and provides a valuable reference for the preparation of high-temperature adsorption resistant functional materials in water-based drilling fluid gel systems.

## 1. Introduction

With the rapid increase in global oil and gas energy demand, and the gradual depletion of shallow oil and gas resources [1,2,3,4,5,6], the efficient development of deep (vertical depth 4500–6000 m) and ultra-deep (vertical depth > 6000 m) oil and gas resources has become an important strategic measure to enhance resource reserves [7,8], alleviate energy shortages, and ensure national energy security and sustainable development [9,10,11,12,13]. Compared with conventional reservoirs, deep oil and gas reservoirs are typically characterized by nonlinear and complex changes in formation pressure [14,15,16,17,18,19], temperature, and other parameters with an increasing drilling depth, leading to more severe operating environments [20,21]. In this context, the problem of wellbore stability under high-temperature conditions is particularly prominent [22]. High temperatures cause desorption between treatment agents and clay, leading to the out-of-control rheology of drilling fluids [23,24,25], increased filtration loss, and subsequent instability accidents such as wellbore instability and diameter reduction [26,27]. Traditional drilling fluid treatment agents mainly rely on non-covalent bonds such as hydrogen bonds and ionic bonds [28,29], but the bond energy of hydrogen bonds and ionic bonds is greatly weakened in the actual working environment of drilling fluids, resulting in high-temperature desorption between treatment agents and clay [30,31,32,33,34].

In recent years, scholars such as Xu et al. [35], Krumpfer et al. [36], Chu Qi et al. [37,38,39], Sun et al. [40], and Zhang et al. [41,42] have successively found that introducing siloxane adsorption groups into the side chains of polymers can significantly improve the high-temperature adsorption stability of polymers. Experiments show that such organosilicon polymers can not only effectively inhibit the hydration swelling of clay, but also have excellent hydrophobic properties. They can absorb the wellbore surface under high-temperature conditions to form a dense polymer hydrophobic film, thereby significantly reducing the negative impact of water-based drilling fluids on wellbore stability. Meanwhile, organosilicon polymers can undergo dehydration condensation with hydroxyl groups on the clay surface to form Si-O-Si covalent bonds [43]. This chemical adsorption mechanism not only strengthens the high-temperature desorption resistance between polymers and clay, but also constructs an isolation film on the clay surface to ensure wellbore stability from both physical and chemical aspects. However, current research still has limitations: most studies focus on the evaluation of the inhibitory performance of film-forming agents [44,45], research on the influence of wellbore characteristics during the film forming process is relatively limited [46,47,48], and there is a lack of systematic analysis of bond types and temperature effects. Most studies only evaluate the inhibition performance at high temperature (>180 °C) and do not systematically explore the mechanism of temperature effect on the adsorption desorption equilibrium of polymers and clay.

In view of the above research status, this paper will deeply analyze the synergistic mechanism of covalent bonds in improving the high-temperature adsorption resistance of polymers through experimental research and theoretical analysis, systematically study the influence of covalent bonds on clay properties under different temperature conditions, and provide a theoretical basis for the research and development of high-temperature inhibitors, and the optimization and construction of deep drilling fluid systems.

## 2. Results and Discussion

### 2.1. Thermal Stability Analysis of Silicone Polymers

**Figure 1:**
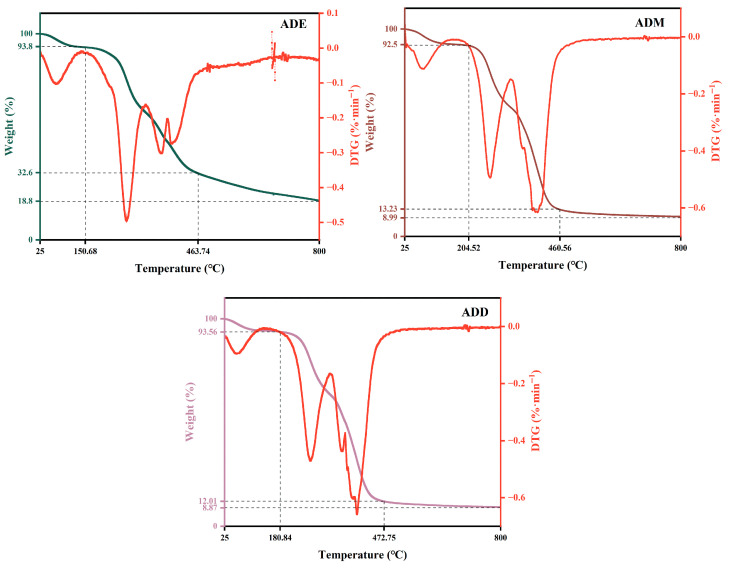
Thermogravimetric curves of organosilicon polymers.

According to the experimental results in Figure 1, the thermal degradation processes of the three organosilicon polymers all show three-stage characteristics. For ADE, from 25.0 to 150.86 °C, there was a weight loss of 6.2% due to the evaporation of surface moisture, with a DTG peak at 68.46 °C. From 150.86 to 463.74 °C, partial thermal cleavage of the main chain led to a weight loss of 61.2%, with DTG double peaks at 318.49 °C and 463.74 °C. From 463.74 to 800 °C, the oxidative decomposition of carbonized residues caused a weight loss of 13.8%. For ADM, from 25.0 to 204.52 °C, there was a weight loss of 7.5% due to moisture evaporation, with a DTG peak at 74.65 °C. From 204.52 to 460.56 °C, dealkylation of quaternary ammonium groups and the hydrolysis of methoxy groups led to main chain fracture, resulting in a weight loss of 79.27%, with a DTG peak at 319.9 °C. From 460.56 to 800 °C, the slow decomposition of residual structures caused a weight loss of 4.24%. For ADD, from 25.0 to 180.84 °C, there was a weight loss of 6.44% due to moisture evaporation, with a DTG peak at 60.16 °C. From 180.84 to 472.75 °C, methyl steric hindrance delays hydrolysis, and main chain oxidation led to a weight loss of 81.55%, with a DTG peak at 353.65 °C. From 472.75 to 800 °C, the decomposition of residual structures caused a weight loss of 5.13%.

The TG-DTG analysis results of the above organosilicon polymers show that before the temperature reached 150–200 °C, there was no obvious mass loss in the samples, proving that the organosilicon polymers themselves have good thermal stability within this temperature range, and their molecular structures are not easily damaged by high temperatures.

### 2.2. Rheological and Filtration Performance Analysis

**Figure 2:**
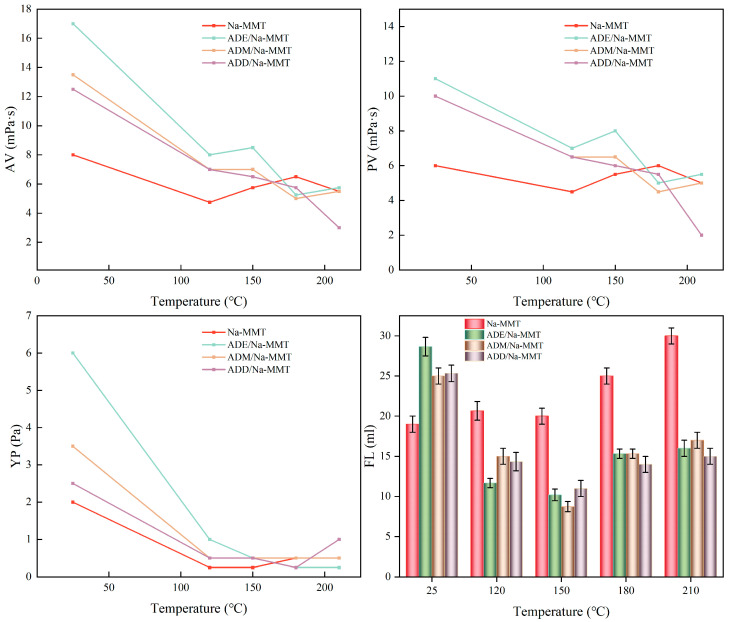
Effect of temperature on the rheological and filtration properties of drilling fluid.

**Figure 3:**
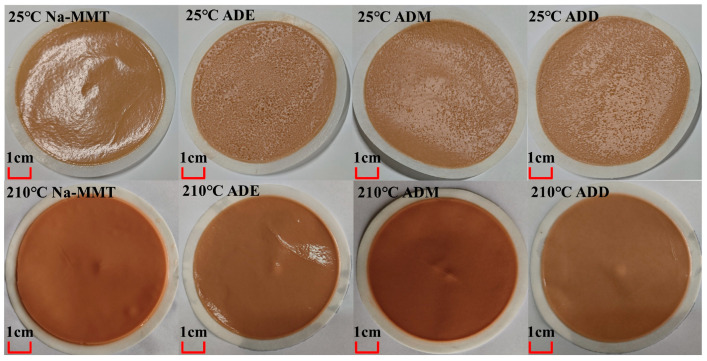
Filter cake images of drilling fluids aged at different temperatures for 16 h.

The rheological parameters (apparent viscosity, AV; plastic viscosity, PV; yield point, YP) and filtration loss (FL_API_) of the drilling fluid gel system were evaluated over a temperature range of 25 °C to 210 °C, with key measurement points at 25 °C, 120 °C, 150 °C, 180 °C, and 210 °C (Figure 2). This range covers the full spectrum of drilling environments, from ambient conditions to ultra-deep high-temperature scenarios, aligning with the core focus on high-temperature adsorption stability. When the temperature rose from ambient to 210 °C, the AV, PV, and YP of all systems significantly decreased, while FL_API_ decreased synchronously. At an ambient temperature, the filtration loss of organosilicon polymer drilling fluid gel systems was generally higher than that of the base slurry system: ADE increased AV by 9 mPa·s, ADM by 5.5 mPa·s, and ADD by 4.5 mPa·s. Specific data are shown in Tables S1–S6. This phenomenon is closely related to the charge neutralization effect dominated by cationic adsorption. As observed in Figure 3, the filter cake at room temperature was thick and loose, showing weak resistance to filtrate penetration, hence the relatively high FL_API_ values (all above 25 mL). However, as the temperature rose to 120–150 °C, FL_API_ exhibited a decreasing trend, reaching the minimum at 150 °C (all below 13 mL), with the formed filter cake being thin and tough (Figure 3). In the 150–210 °C range, FL_API_ rebounded to above 14 mL. This aligns with [49], which emphasizes chemical cross-linking as the driver of long-term rheological stability in high-temperature colloidal systems, rather than physical adsorption alone.

In summary, the FL_API_ of organosilicon polymer drilling fluid gel systems significantly decreased at high temperatures, and the filter cake transformed from thick to thin with toughness. This is attributed to the transition of adsorption between organosilicon polymers and clay from electrostatic adsorption to covalent bond adsorption as the temperature rises.

### 2.3. Shale Rolling Recovery of Organosilicon Polymer

**Figure 4:**
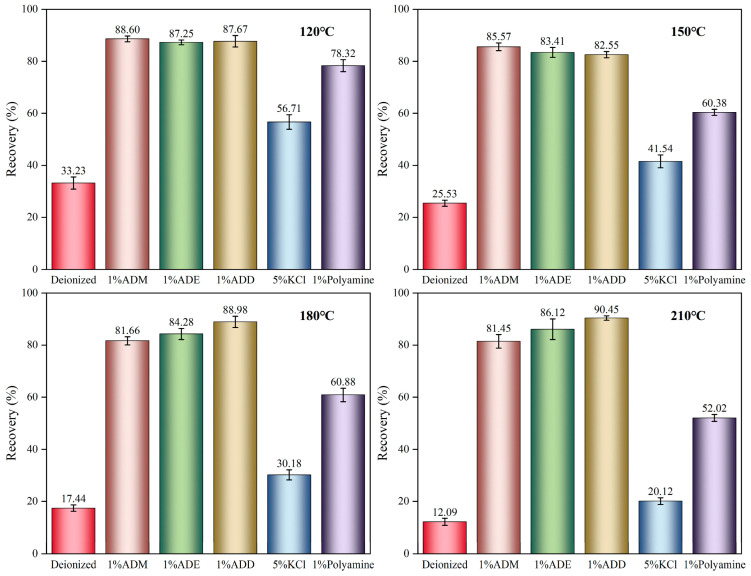
A comparison of data from the 16 h rolling recovery tests on shale cuttings using three organosilicon polymer inhibitors and common inhibitors at different temperatures. Specific data are shown in Tables S6–S9.

The high-temperature resistance of shale inhibitors is crucial for borehole stability. The shale rolling rate test evaluated the ability of inhibitors to prevent clay hydration and dispersion under dynamic thermal conditions, directly reflecting their potential to maintain wellbore stability in high-temperature drilling [50]. High temperature accelerates shale hydration by enhancing the kinetic energy of water molecules, leading to clay expansion and microcrack propagation; this process weakens the integrity of shale and reduces the rolling recovery rate [51]. Organosilicon polymers alleviate this situation as follows: as shown in Figure 4, the rolling recovery rates of organosilicon polymers within the temperature range of 120 °C to 210 °C were all higher than those of polyamine inhibitors and KCl, demonstrating that organosilicon polymers have a significant effect in inhibiting the hydration dispersion of shale cuttings at high temperatures. It is speculated that the siloxyl groups in the polymer molecules can undergo dehydration condensation with the silanol groups in the base slurry to form covalent bonds in an aqueous solution. This allows organosilicon polymers to adsorb firmly on clay surfaces, prevent further hydration, and maintain a high recovery rate even at 210 °C. Meanwhile, within the temperature range of 120 °C to 150 °C, the shale rolling recovery rates of ADM were 88.6% → 85.57%, higher than those of ADE (87.25% → 83.41%) and ADD (87.67% → 82.55%). In the range of 150 °C to 210 °C, the shale rolling recovery rates of ADD (88.98% → 90.45%) and ADE (84.28% → 86.12%) were both higher than that of ADM (81.66% → 81.45%).

### 2.4. Fourier Transform Infrared Spectroscopy (Ft-Ir) Spectroscopy Analysis

**Figure 5:**
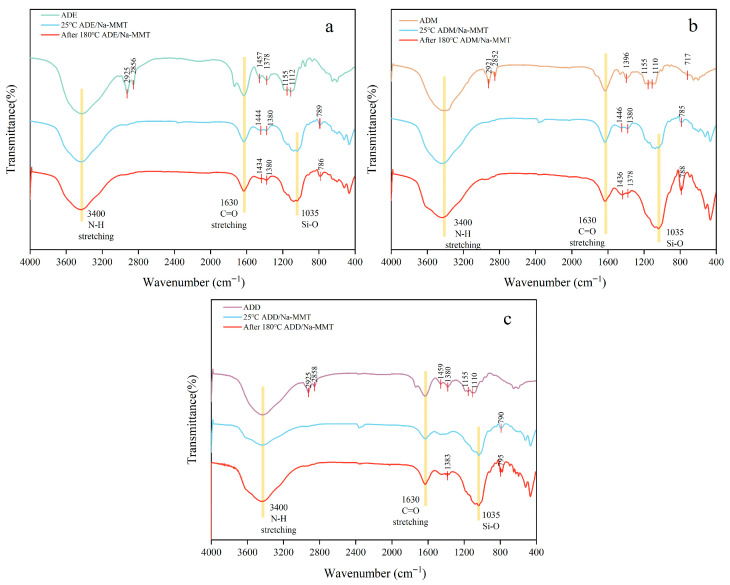
FT-IR Spectra of organosilicon polymers before and after high-temperature adsorption with clay ((**a**) is polymer ADE, (**b**) is polymer ADM, and (**c**) is polymer ADD).

According to Table 1 and Figure 5, polymers ADE, ADM, and ADD were successfully synthesized. Additionally, if siloxyl groups in organosilicon monomers undergo hydrolysis and self-condensation during emulsion polymerization, Si-O-Si bonds would form, characterized by strong broad absorption peaks of equal intensity at 1020 cm^−1^ and 1035 cm^−1^ (symmetric stretching vibrations), and an obvious bending vibration peak at 800 cm^−1^. However, no such characteristic peaks were observed at 1020 cm^−1^, 1035 cm^−1^, or 800 cm^−1^ in the FT-IR spectra of organosilicon polymers (Figure 5), confirming that organosilicon monomers did not hydrolyze or condense during polymerization [17].

By comparing the infrared spectrum curves of the organosilicon polymer mixed with clay in Figure 5, it is found that at room temperature, an obvious peak representing the Si-O-Si bond was observed at approximately 1035 cm^−1^ in the curve of the polymer mixed with clay. However, the peak intensity at 1035 cm^−1^ in the curve of the polymer mixed with clay after high-temperature aging was significantly higher than that in the curve of the polymer mixed with clay at room temperature. According to the Lawbert–Beer law, A = Lg (1/T) = Kbc (that is, absorbance A is proportional to the substance concentration c of the medium and the absorption layer thickness b). From qualitative analysis, the absorption intensity of the spectral peaks in the infrared spectrum reflected the concentration of functional groups to a certain extent [25]. Therefore, it is analyzed that high temperature promoted the adsorption between clay and organosilicon polymers, so that the organosilicon polymers covered the surface and interlayers of bentonite.

### 2.5. Effect of Different Temperatures on the Zeta Potential and Particle Size Distribution of the Drilling Fluid Gel System

**Figure 6:**
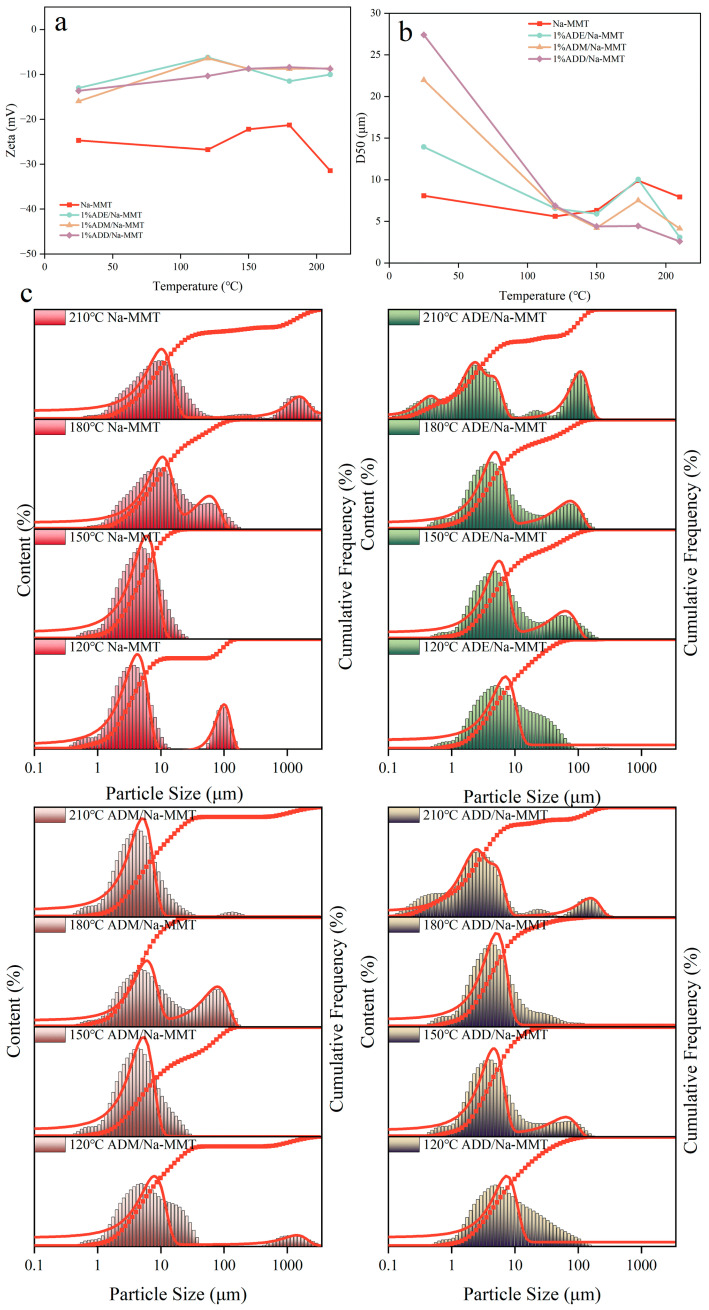
Effect of temperature on the zeta potential (**a**), D50 (**b**), and particle size distribution (**c**) of the drilling fluid gel system.

As shown in Figure 6a,b, organosilicon polymers are positively charged, while clay particles are negatively charged. At 25 °C, organosilicon polymers and clay undergo neutralization and adsorption due to electrostatic interactions, resulting in the particle size of the system with organosilicon polymers added being larger than that of pure clay particles at room temperature. At ambient temperature, the quaternary ammonium groups of polymers neutralize the negative charges of clay, reducing the absolute value of the zeta potential by 44–50%. Charge neutralization weakens the electrostatic repulsion between particles. In the 25–120 °C stage, the high-temperature dispersion of clay dominated, leading to a gradual decrease in D50: the D50 of the base slurry decreased from 8.07 μm to 5.605 μm; for ADE, the zeta potential increased from −13 mV to −6.2 mV, and D50 decreased from 27 μm to 6.55 μm; for ADM, the zeta potential increased from −16 mV to −6.4 mV, and D50 decreased from 27.4 μm to 6.69 μm; for ADD, the zeta potential increased from −13.7 mV to −10.4 mV, and D50 decreased from 21.98 μm to 6.87 μm. In the 120–180 °C stage, the base slurry aggregated due to intensified thermal motion, causing D50 to increase, but the polymer system inhibited aggregation due to covalent bond crosslinking, resulting in a D50 growth rate smaller than that of the Na-MMT curve. In the 180–210 °C stage, the high-temperature dispersion of clay dominated again, leading to a gradual decrease in D50. Electrostatic adsorption between polymers and clay increased the particle size, corresponding to thicker filter cakes and higher filtration loss (Figure 2 and Figure 3).

Meanwhile, the curves of ADE/Na-MMT and ADD/Na-MM at 120 °C broadened to 1–100 μm, with a significant secondary peak in ADE (>10 μm), as shown in Figure 6c. In the 120–180 °C stage, the base slurry was dominated by aggregation, while the polymer drilling fluid gel system inhibited aggregation, speculated to be due to covalent bonds tightly adsorbing onto the clay surface. ADM formed limited covalent bonds due to the early hydrolysis depletion of groups, failing to completely wrap the clay surface, so high-temperature aggregation caused particle size distribution to shift rightward, forming bimodal peaks. In the 180–210 °C range, the high-temperature dispersion of clay prevails and ADM’s particle size distribution tended to concentrate.

Based on changes in zeta potential data and particle size distribution, the adsorption mechanism was inferred to shift from physical adsorption (charge neutralization) to chemical adsorption (covalent bonding). The adsorption layer regulated the aggregation-dispersion state of clay particles: charge neutralization dominated particle coagulation at low temperatures, while covalent crosslinking and hydrophobic film formation inhibited clay hydration swelling at high temperatures.

### 2.6. Effect of High Temperature on the Polymer Adsorption and Conductivity of the Polymer Drilling Fluid Gel System

**Figure 7:**
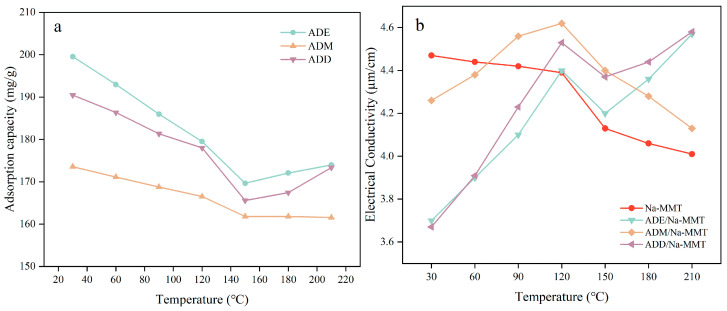
Adsorption properties of silicone polymer at different temperatures (**a**), and conductivity changes of the silicone polymer drilling fluid gel system (**b**).

In the silicone polymer drilling fluid gel system, changes in conductivity essentially reflect variations in free ion concentration and are directly linked to the hydrolysis process of the polymer. As shown in Figure 7, within the temperature range of 30 ° C to 150 ° C, the adsorption capacity of all three drilling fluid gel systems showed a downward trend, attributed to the gradual desorption of electrostatic adsorption (hydrogen bonds/ionic bonds) formed at low temperatures under high-temperature conditions. Among them, the ADM polymer drilling fluid gel system exhibited the highest conductivity at room temperature (4.26 μS/cm) due to the high activity of methoxy groups, which rapidly hydrolyzed at room temperature to generate abundant silanol and alcohol [26], significantly increasing free ion concentration. However, its adsorption capacity was only 173.57 mg/g because the partial self-polymerization of Si-OH groups prevented them from participating in bond formation. In the 30–120 °C range, the conductivity of the ADM polymer drilling fluid gel system increased from 4.26 μS/cm to 4.62 μS/cm, while in the 120–210 °C range, conductivity decreased to 4.13 μS/cm and adsorption capacity dropped to 161.55 mg/g. This occurred because the rapid hydrolysis of methoxy groups in ADM increased free ion concentration, but high temperatures exhausted ADM’s active groups, interrupting bond formation.

In the 150–210 °C range, the conductivity of ADE and ADD polymer drilling fluid gel system increased (ADE: 4.2 μS/cm → 4.57 μS/cm, ADD: 4.37 μS/cm → 4.58 μS/cm), accompanied by a synchronous increase in adsorption capacity (ADE: 169.65 mg/g → 173.97 mg/g, ADD: 165.57 mg/g → 173.36 mg/g). This resulted from the slow hydrolysis of ethoxy groups and methyl steric hindrance inhibiting self-polymerization, allowing for Si-OH to continuously form Si-O-Si covalent bonds with clay at high temperatures. Although the partial self-polymerization of Si-OH in ADM led to fewer covalent bonds with clay, the remaining bonds still firmly adsorbed the treatment agent, maintaining an adsorption capacity of 161 mg/g between 150 °C and 210 °C. The inflection points in conductivity and adsorption capacity at 150 °C were corroborated by the synchronous changes in the rolling recovery rate at 150 °C in Figure 4, confirming that the inhibitory performance of polymers depends on the hydrolysis rates of substituents.

In conclusion, when the temperature exceeded 150 °C, organosilicon polymers maintained strong adsorption to clay through Si-O-Si covalent bonds, showing no obvious desorption, which verifies the advantage of covalent bonds in resisting high-temperature desorption.

### 2.7. Effects of High Temperature on the Microscopic Morphology and Elemental Composition of the Polymer–Shale Surface Film

**Figure 8:**
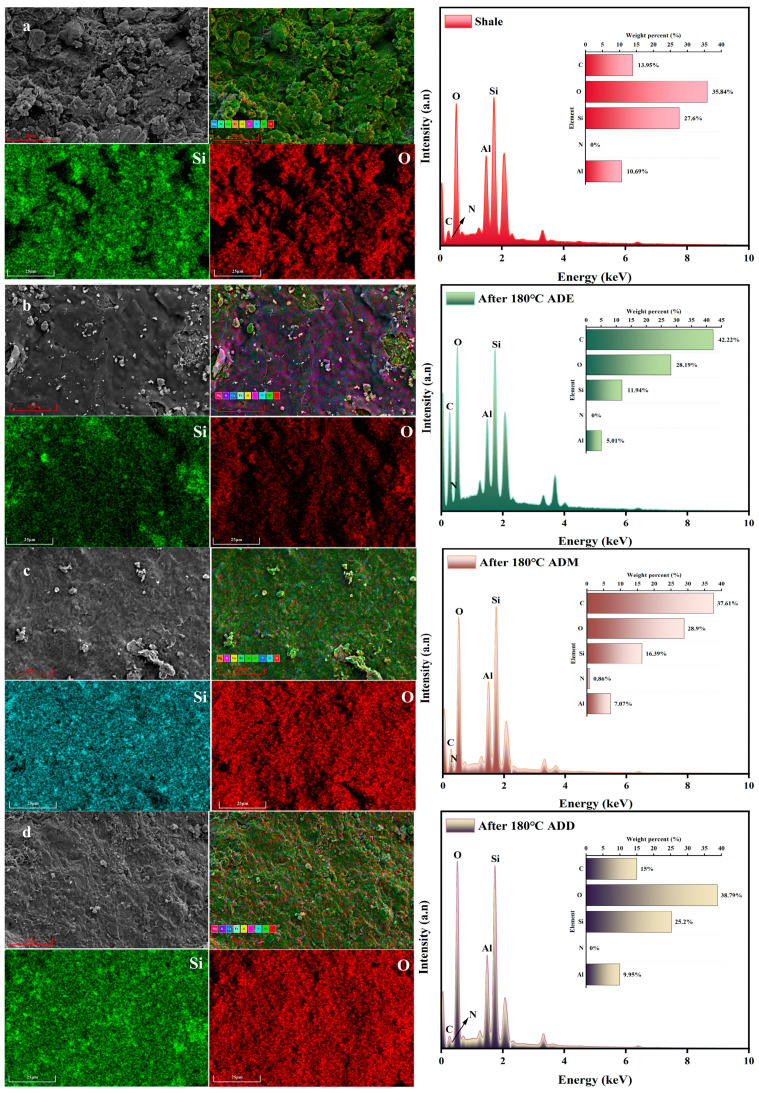
Microscopic morphology and elemental analysis comparison of the shale surface and original shale sample after aging in a 1% organosilicon polymer solution at 180 °C for 16 h. Distribution of other elements is shown in Figures S1–S4 and elemental content is extracted from Tables S10–S13. (**a**) Shale samples; (**b**) ADE polymer high-temperature modified shale cuttings; (**c**) ADM polymer high-temperature modified shale cuttings; (**d**) ADD polymer high-temperature modified shale cuttings.

As shown in Figure 8a, the original shale exhibited a well-developed bedding structure, with abundant microcracks and pores observable on the surface. In contrast, the micro morphologies of shales modified by organosilicon polymers (Figure 8b–d) show significantly smoother and flatter surfaces, where obvious micro-pores and microcracks disappear. A translucent polymer film was clearly visible on the surface, indicating that after adsorption, the organosilicon polymer formed a dense polymer film on the shale surface, covering the original texture of the shale.

After adsorption modification with the solution, the normalized mass percentage of carbon elements on the shale surface in Figure 8b increased from 13.95% to 42.22%, the oxygen content decreased from 35.84% to 28.19%, and the silicon content decreased from 27.6% to 16.39%. Compared with the untreated shale surface, the carbon element ratio of the modified shale sample in Figure 8b increased significantly, while the oxygen and silicon contents decreased. The elemental ratio on the modified shale surface was close to the theoretical value of the elemental mass composition of the polymer, suggesting that the thin film formed on the shale surface was adsorbed by organosilicon polymers.

Following solution adsorption modification, the normalized mass percentage of carbon on the shale surface in Figure 8c increased from 13.95% to 37.61%, oxygen content decreased from 35.84% to 28.9%, and silicon content dropped from 27.6% to 16.39%. Compared with the untreated shale, the modified sample in Figure 8c showed a significant rise in carbon proportion, alongside reduced oxygen and silicon contents. The elemental ratios on the modified surface align with the theoretical mass composition of the polymer, confirming that the formed film resulted from organosilicon polymer adsorption. This finding is consistent with the adsorption test results presented in Figure 7.

After the shale surface in Figure 8d was modified by solution adsorption, the normalized mass percentage of carbon elements on the sample surface increased from 13.95% to 15%, the normalized mass percentage of oxygen elements changed from 35.84% to 38.79%, and the normalized mass percentage of silicon elements changed from 27.6% to 25.2%. Compared with the surface of the untreated shale sample, the contents of carbon and oxygen elements on the surface of the modified shale sample in Figure 8d were basically the same, and the content of silicon elements was slightly decreased. The reason is that ADD contains two methoxy groups, while the control group (ADM/ADE) contains three siloxy groups. The three siloxy groups in the control group hydrolyzed to generate three silanol groups, forming a denser three-dimensional crosslinked network that enhanced chemical bonding with clay. Additionally, the methyl group in VMDS exhibited strong hydrophobicity, causing the polymer to enrich on the surface of shale cuttings and form a dense hydrophobic film. The thickness of this film may exceed the detection depth of surface elemental analysis, leading to the failure to detect carbon elements in the shale matrix. In contrast, the crosslinked network of trisiloxy groups in the control group was more open, allowing for organic segments to be more easily exposed on the surface, resulting in obvious carbon signals.

Experimental results confirm that the organosilicon polymer reacted with and adsorbs onto the shale surface. Even after high-temperature aging at 180 °C, the organosilicon polymer remained adsorbed on the shale surface without desorption. Therefore, organosilicon polymers can still exert a strong inhibitory effect on the hydration and dispersion of shale at high temperatures.

### 2.8. Wettability Analysis

**Figure 9:**
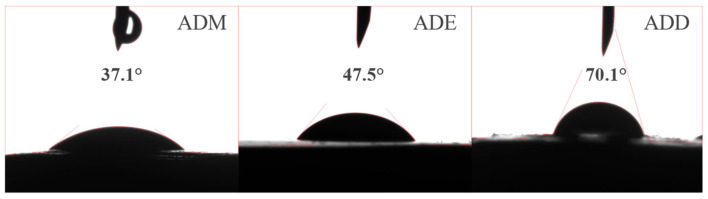
Comparison of the contact angles of different polymers after high temperature at 180 °C.

As observed from the contact angle experiment in Figure 9, after forming a film with the shale core using the ADD polymers, there was certain hydrophobic property, while the films formed by using the ADM and ADE polymers with the shale core showed poor hydrophobicity. The contact angle of ADD was 70.1°, indicating the strongest hydrophobicity, which is attributed to the methyl steric hindrance and dimethoxy groups in VMDS. The methyl group, as a strong hydrophobic group, directly enhanced the hydrophobicity of the film’s surface. After hydrolysis, the dimethoxy groups formed dual-site Si-O-Si bonds. Although the crosslinking density was lower than that of the trimethoxy groups, the steric hindrance restricted the self-polymerization of methoxy groups in water, and the methyl groups were directionally arranged on the film surface to form a hydrophobic barrier. The contact angle of ADM was 37.1°, showing the weakest hydrophobicity, as the quaternary ammonium groups had strong hydrophilicity, leading to high hydrophilicity of the film surface. The contact angle of ADE was 47.5°, with hydrophobicity between the two, because the ethoxy group itself contained a hydrophilic ethyl chain, which weakens the hydrophobic effect.

These experimental phenomena indicate that polymers with -CH_3_ steric hindrance substituents can form hydrophobic films on the shale surface. At high temperatures, their inhibitory performance was better than that of the other two organosilicon polymers (Figure 4).

## 3. Proposed Inhibition Mechanism

**Figure 10:**
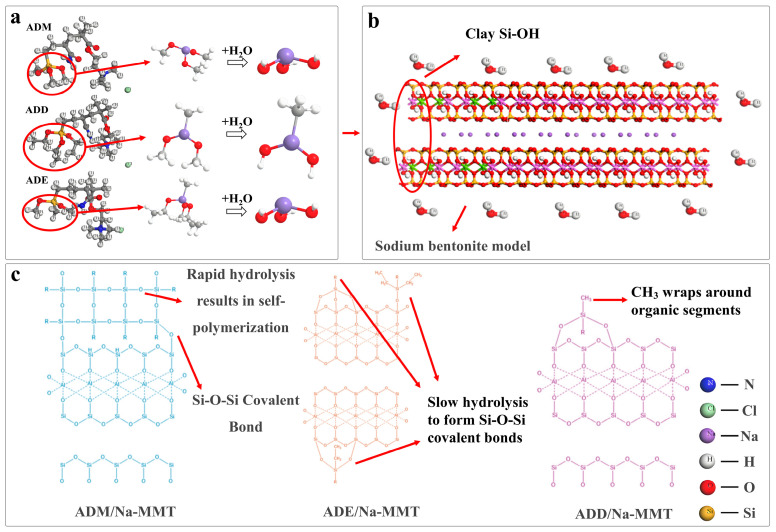
Adsorption mechanisms of different groups with clay at high temperature. (**a**) The hydrolysis process of three types of organic silicon monomers; (**b**) Organic silicon adsorbs with clay to form covalent bond sites; (**c**) Molecular structure of Si-O-Si covalent bonds formed by the adsorption of three types of organic silicon monomers with clay.

Based on the above experiments, the synergistic mechanism by which covalent bonds enhance the high-temperature adsorption performance of clay can be inferred: the temperature drives the adsorption mechanism to transition from physical adsorption dominated by quaternary ammonium groups at low temperatures to chemical adsorption dominated by Si-O-Si covalent bonds at high temperatures. After hydrolysis of the siloxyl groups in organosilicon polymers, Si-OH (Figure 10a) forms Si-O-Si covalent bonds with the Si-OH sites on the clay surface of sodium-based bentonite (Figure 10b). It is found that substituents synergistically regulate bonding efficiency through hydrolysis rate and steric hindrance: trimethoxy groups hydrolyze rapidly but self-polymerize, resulting in weak high-temperature adsorption capacity. Ethoxy groups hydrolyze slowly to form a dense covalent network. Dimethoxy groups with methyl steric hindrance delay hydrolysis, while methyl groups enrich on the shale surface. The polymer forms a dual-site crosslinked film at high temperatures, exhibiting certain hydrophobicity (Figure 10c).

This mechanism reveals the synergistic law of “temperature-group-covalent bond”, in which temperature affects the degree of group hydrolysis, the degree of group hydrolysis restricts the formation of covalent bonds with clay, and the formation of covalent bonds is closely linked to the excellent high-temperature resistance.

## 4. Conclusions

(1) In this study, three organosilicon polymer inhibitors were successfully synthesized via soap-free emulsion polymerization. Experiments show that their rolling recovery rate at 210 °C was 30% higher than that of polyamine and KCl (exceeding 80%). After hydrolysis, the siloxane groups of the polymer generated Si-OH, which underwent dehydration condensation with Si-OH on the clay surface to form Si-O-Si covalent bonds. This resulted in a denser filter cake and a 53.3% reduction in fluid loss (decreasing to 14 mL), confirming that the covalent bond adsorption mechanism significantly enhanced the high-temperature stability of clay compared to traditional non-covalent bonds (hydrogen bonds/ionic bonds).
(2) At room temperature, electrostatic adsorption occurred between the polymer and clay (the absolute value of zeta potential of the polymer drilling fluid gel system decreases by 44–50% after adding the polymer). The infrared spectrum of the polymer-clay mixed sample aged at 180 °C showed an increased peak of Si-O bonds, proving the formation of covalent bonds. This indicates that the adsorption between the polymer and clay shifted from low-temperature electrostatic adsorption to a synergistic effect of high-temperature electrostatic adsorption and covalent bond adsorption.
(3) Experiments on conductivity and adsorption amount demonstrate that different substituent types affect the hydrolysis rate of siloxane groups, thereby determining the thermal stability of the covalent bonds formed between the polymer and clay at high temperatures: methoxy groups hydrolyzed rapidly, but were prone to self-polymerization and depletion at high temperatures, leading to bond disruption. Ethoxy groups maintained hydrolysis activity and continuously formed bonds at high temperatures. Methyl siloxyl groups with steric hindrance could inhibit the self-polymerization of methoxy groups, and the formed film exhibited certain hydrophobicity (contact angle: 70.1°).
(4) In the future, drilling fluid conditioner types can be optimized: ADD-type polymers with steric hindrance are suitable for ultra-high temperature environments, combining inhibitory and fluid loss reduction properties. Meanwhile, introducing multifunctional groups (such as the synergy of amino and methoxy groups) can regulate hydrolysis timing and hydrophobicity, or compounding with nano-silica can enhance film strength to further improve high-temperature stability. Exploring biobased siloxane monomers to reduce synthesis costs and environmental toxicity will promote the development of green drilling fluid technology.

## 5. Experimental

### 5.1. Experimental Reagents

(1) Main chemical reagents

Methacryloyloxyethyl trimethyl ammonium chloride (DMC), vinyl methyldimethoxysilane (VMDS), vinyl trimethoxysilane (VTMO), and vinyl triethoxysilane (VTES) (analytical grade) were purchased from Shanghai Macklin Biochemical Technology Co., Ltd., Shanghai, China). Acrylamide (AM) (analytical grade) was purchased from Aladdin Biochemical (Shanghai) Co., Ltd., Shanghai, China. Span 80 and Tween 80 (analytical grade) were purchased from Sinopharm Group Co., Ltd., Shanghai, China. Sodium bentonite (industrial grade) was purchased from Weifang Huawei Bentonite Co., Ltd., Weifang, China. Potassium chloride (analytical grade) was purchased from Shanghai Hushi Chemical Co., Ltd., Shanghai, China. Absolute ethanol (analytical grade) was purchased from Shanghai Macklin Biochemical Technology Co., Ltd., Shanghai, China. Finally, 2,2-azobis (2-methylpropionitrile) (ABIN, 98%) was purchased from Shanghai Macklin Biochemical Technology Co., Ltd., Shanghai, China.

(2) Analysis of the mineral composition of shale rock fragments

The shale rock debris rolling recovery experiment used outcrop rock debris shale samples provided by China Petroleum Chuanqing Drilling Engineering Co., Ltd., Chengdu, China The mineral composition of shale rock fragments was determined using INCA-X-ray diffraction (XRD), and the mineral composition analysis results of shale samples are shown in Table 2.

### 5.2. Experimental Instruments

A Thermo ElECTRON CORPORATION (5700) infrared spectrometer (Shimadzu, Japan) was used, along with a TGA 2 thermogravimetric analyzer (Mettler-Toledo, Shanghai, China), BGRL-2 roller heating furnace (Qingdao Tong Chun Petroleum Instruments Co., Ltd., Qingdao, China), ZEISS EVO LS 15 scanning electron microscope (Carl Zeiss Microscopy, Germany, Jena), WT-HTHP-50 SL high-temperature and high-pressure rheometer (Beijing Prospecting Engineering Research Institute, Beijing, China), and GX3020GF20 electrothermal constant-temperature drying oven (Guangdong Gaoxin Instruments Co., Ltd., Guangzhou, China).

### 5.3. Preparation of Organosilicon Polymer

To prevent hydrolysis during synthesis, organosilicon polymers were prepared via emulsion polymerization. The required points for synthesis are shown in Table 3.

### 5.4. Thermogravimetric Analysis

The thermal stability of the polymer samples was investigated using a TGA-2 thermogravimetric analyzer (METTLER TOLEDO, Greifensee, Switzerland). The test parameters were set as follows: temperature range 30.0–800.0 °C, heating rate 10.0 K·min^−1^, and nitrogen as the protective inert gas with a flow rate of 20.0 mL·min^−1^. The TG-DTG curves of organosilicon polymers were obtained from the experiments.

### 5.5. Rheological Filtration Experiment

Then, 0.70 g of anhydrous sodium carbonate was added to 350 mL of water, stirred until completely dissolved, and then 14.00 g of sodium bentonite added. This was stirred with an electric mixer for 2 h, and then aged hermetically at room temperature for 24 h to form a fresh water base slurry with a soil content of 4%.

The apparent viscosity (AV), plastic viscosity (PV), and yield point (YP) of the drilling fluid were measured using a six-speed rotational viscometer. The API filtration loss of the drilling fluid gel system was measured using an API medium-pressure filtration loss instrument. The rheological parameters of the drilling fluid were calculated according to API standards.

AV = Φ600/2 (mPa∙s)(1)

PV = Φ600 − Φ300 (mPa∙s)(2)

YP = 0.48 × (Φ300 − PV) (Pa)(3)

### 5.6. Evaluation of Rolling Recovery Performance

Twenty grams of shale cuttings (6–10 mesh) and 350 mL of organosilicon polymer solution (1% concentration) were placed in an aging tank and then aged for 16 h in a BGRL-2 roller heating furnace at a rolling speed of 30 r/min within the temperature range of 120–210 °C under atmospheric pressure, as this experiment focused on isolating the effect of temperature on the adsorption stability of organosilicon polymers to clarify the mechanism by which covalent bonds enhance clay–polymer adsorption under high-temperature conditions, while high-pressure effects involve complex factors requiring independent experimental design. Therefore, the influence of pressure on organic silicon polymers was not considered.

After cooling to room temperature, the shale cuttings were screened through a 40-mesh sieve, and the remaining cuttings were rinsed with deionized water to remove residues. The rinsed cuttings were dried in a vacuum drying oven at 105 ± 3 °C for 24 h until constant weight, weighed, and the mass of the cuttings sample was recorded as M. The experiment was repeated three times to take the average value. The calculation of shale rolling recovery rate is shown in Equation (4).

R = M/20 × 100%(4)

R—rolling recovery rate of shale, %;
M—weight of dried cuttings after aging, g.

### 5.7. Infrared Spectroscopy Analysis

The organosilicon polymer was purified with absolute ethanol and acetone, dried in an oven at 60 °C, taken out, crushed, and sieved through a 100-mesh sieve to obtain white powdery granular products for infrared spectroscopy analysis.

Then, 1.0 wt% organosilicon polymer was added to the 4.0 wt% sodium bentonite suspension, which was then placed in an aging tank and aged at 180 °C for 16 h. Subsequently, it was vacuum-dried at 105 °C to constant weight, crushed, and sieved through a 200-mesh sieve to prepare modified soil samples for infrared spectroscopy analysis.

### 5.8. Zeta Potential Experiment

The zeta potential of nanoemulsions was measured using a Zetasizer Nano ZS90 series nanosize potentiometer. The specific procedures were as follows: sodium montmorillonite (MMT) and deionized water were mixed at a mass ratio of 1:100 to prepare 10 mL of solution, which was stirred with a high-speed stirrer at 8000 rpm for 20 min to form a fully dispersed bentonite suspension. Four portions (10 mL each) of the clay dispersion were taken, and equal volumes (10 mL) of four organosilicon polymer (1 wt% concentration) were added, respectively. After mechanical stirring for 30 min, the samples were divided into aging cells, sealed, and placed in a high-temperature oven. The aging temperature was set at 120–210 °C (simulating extreme thermal environments), and the aging time was 16 h. The control group was unaged samples (stored at room temperature). After 16 h, the aged samples were taken out, cooled to room temperature, and mechanically stirred for 30 min to restore particle dispersibility and prevent agglomeration from interfering with the test. The nanoemulsion to be tested was then dropped into a cuvette, which was placed in the instrument for testing.

### 5.9. Particle Size Experiment

The Mastersizer 3000 laser particle size analyzer was operated. After setting the sample parameters and measuring the deionized water background, a small amount of polymer drilling fluid aged at 120–210 °C, as described in Section 5.8, was dropped into a sample tank filled with deionized water, fully dispersed at a rotation speed of 2700 r·min^−1^, and the particle size distribution was measured three times. The average value was taken to obtain the particle size distribution of the drilling fluid gel system.

### 5.10. Total Organic Carbon Analysis (TOC) Adsorption Experiment

The experiment first involved preparing 100 mL of 4% sodium bentonite, then adding 1.0% organosilicon polymer, stirring at 25 °C for 1 h, and heating at different temperatures for 2.5 h. Equal volumes of the organosilicon polymer/sodium bentonite mixture were taken and centrifuged (4000 rpm for 30 min). The supernatant was collected with a pipette, diluted to a concentration range of 10–100 ppm, and the total organic carbon (TOC) content was measured. The adsorption capacity of the polymer on the clay surface was calculated using Equation (5), and the adsorption curve was plotted.

(5) $$T=\frac{C_{J}-\frac{C_{S}}{\omega }}{C_{T}}$$

T—adsorption capacity, mg·g^−1^;
C_J_—polymer concentration, mg·L^−1^;
C_S_—total organic carbon concentration in the supernatant, mg·L^−1^;
ω—mass fraction of the carbon element in the polymer;
C_T_—concentration of clay particles in the dispersion system, g·L^−1^.

### 5.11. Measuring the Degree of Hydrolysis by Conductivity

First, 1% of self-made polymers ADE, ADM, ADD, and AD were added into four cups of 350 mL 4% bentonite base slurry, respectively. The mixtures were stirred with a high-speed stirrer at 8000 rpm for 20 min. After the mixed solutions were fully dispersed, they were transferred to a high-temperature reactor and heated at different temperatures for 2.5 h. The mixed solutions were taken out and stirred at a high speed. After the mixed solutions were evenly stirred, their conductivity was measured.

### 5.12. Scanning Electron Microscopy (SEM) and Energy-Dispersive X-Ray Spectroscopy Analysis (EDS)

The shale samples were placed into a reaction kettle containing 350 mL of 1% polymers, and subjected to the film forming reaction under rolling circulation at 180 °C for 16 h. After the reaction, the heating furnace was allowed to cool to room temperature, and the shale samples in the reaction kettle were taken out. The samples were rinsed with flowing tap water to remove the nanoemulsion and nanoparticles attached to the surface, and air-dried at room temperature. The micro-morphologies of the original shale surface and the shale surfaces modified by several polymer solutions were studied by a ZEISS EVO LS 15 scanning electron microscope (Carl Zeiss Microscopy, Jena, Germany). In addition, the chemical elements on the surface of shale cuttings were analyzed by EDS spectroscopy.

### 5.13. Contact Angle Experiment

A 3% organosilicon polymer solution was prepared, shale cores immersed in the solution, and aged at 180 °C for 16 h. Then, it was removed and dried in a vacuum oven at 60 °C to constant weight. Using a contact angle goniometer, a 5.0 μL deionized water droplet was dispensed onto the surface of different samples with a microsyringe. Standing for 2 min, the rock surface and droplet morphology were magnified via measurement software. Droplet images were captured under light source irradiation using the instrument’s built-in camera, and the contact angles were measured and recorded.

## Figures and Tables

**Table 1:** Analysis of infrared spectral peaks of organosilicon polymers.

Absorption Peak Position	Analysis	Intensity
3400 cm^−1^	N-H stretching vibration in AM	Strong
2800–2975 cm^−1^	C-H stretching vibration	Weak
1630 cm^−1^	Characteristic absorption peak of C=O stretching vibration	Strong
1460 cm^−1^	C-N stretching vibration in DMC	Weak
1380 cm^−1^	N-CH_3_ stretching vibration in DMC	Weak
1155 cm^−1^	C-O stretching vibration	Moderate
1110 cm^−1^, 1112 cm^−1^	Characteristic peaks of Si-O bonds in VTES, VTMO, VMDS	Moderate

**Table 2:** The results of mineralogical composition analysis of the shale samples.

Component	Content (wt%)	Clay Mineral Component	Content (wt%)
Quartz	36.5	Kaolinite	1.3
Potassium Feldspar	4.3	Chlorite	8.6
Sodium Feldspar	4.4	Illite	13.7
Calcite	8.2	Illite/Smectite Mixed Layer	20.2
Dolomite	2.8		

**Table 3:** Preparation process of the organic silicon polymer.

Step	Added Reagents	Ratio	Stirring Speed (r/min)	Stirring Time (min)	Function
1	AM, DMC	3:1	500	30	Pre-dissolve the two monomers in deionized water to obtain Solution A.
2	C_2_H_4_O_2_, NaOH		500	20	Add saturated sodium hydroxide solution and glacial acetic acid to Solution A to adjust the pH value.
3	Span 80, Tween 80	3:1	600	30	Prepare the emulsifier.
4	VTES	5%	500	20	Pre-dissolve the organosilicon monomer VTES in absolute ethanol to obtain organosilicon monomer Solution B.
5	ABIN	0.5%	200	20	Transfer the emulsifier and Solution B to a three-necked flask equipped with a nitrogen inlet, condenser, and magnetic stirrer. Add ethanol (1 wt%) as a hydrolysis inhibitor and preheat the mixture to the reaction temperature. Then, introduce the initiator ABIN and carry out the reaction under constant temperature stirring to obtain polymer ADE. Replace the organosilicon monomer in Step 4 with VTMO and VMDS to obtain polymers ADM and ADD, respectively.

## Data Availability

The data presented in this study are openly available in the article.

## Supplementary Materials

The following supporting information can be downloaded at: https://www.mdpi.com/article/10.3390/gels11080623/s1, Table S1: Rheological filtrate loss data of different 1% polymers added to a 4% base slurry at 25 °C; Table S2: Rheological filtrate loss data of different 1% polymers added to a 4% base slurry at 120 °C; Table S3: Rheological filtrate loss data of different 1% polymers added to a 4% base slurry at 150 °C; Table S4: Rheological filtrate loss data of different 1% polymers added to a 4% base slurry at 180 °C; Table S5: Rheological filtrate loss data of different 1% polymers added to a 4% base slurry at 210 °C; Table S6: The data results of three organosilicon polymer inhibitors and commonly used inhibitors subjected to 16-h rolling aging on shale cuttings at 120 °C, with measurements taken three times and averaged; Table S7: The data results of three organosilicon polymer inhibitors and commonly used inhibitors subjected to 16-h rolling aging on shale cuttings at 150 °C, with measurements taken three times and averaged; Table S8: The data results of three organosilicon polymer inhibitors and commonly used inhibitors subjected to 16-h rolling aging on shale cuttings at 180 °C, with measurements taken three times and averaged; Table S9: The data results of three organosilicon polymer inhibitors and commonly used inhibitors subjected to 16-h rolling aging on shale cuttings at 210 °C, with measurements taken three times and averaged; Table S10: The content of other elements on the surface of shale after rolling aging at 180 °C for 16 h; Table S11: The content of other elements on the surface of shale after being treated with a 1% ADE polymer solution at 180 °C for 16 h of rolling aging; Table S12: The content of other elements on the surface of shale after being treated with a 1% ADM polymer solution at 180 °C for 16 h of rolling aging; Table S13: The content of other elements on the surface of shale after being treated with a 1% ADD polymer solution at 180 °C for 16 h of rolling aging; Figure S1: Distribution of other elements on the shale surface after rolling aging for 16 h at 180 °C; Figure S2: Distribution of other elements on the shale surface modified by 1% ADE polymer solution after rolling aging at 180 °C for 16 h; Figure S3: Distribution of other elements on the shale surface modified by 1% ADM polymer solution after rolling aging at 180 °C for 16 h; Figure S4: Distribution of other elements on the shale surface modified by 1% ADD polymer solution after rolling aging at 180 °C for 16 h.

## Abbreviations

WBDFs	Water-based drilling fluids
SEM	Scanning electron microscopy
mm	Millimeter
TOC	Total organic carbon analysis
EDS	Energy-dispersive X-ray spectroscopy
Na-MMT	Sodium bentonite
AM	Acrylamide
DMC	Methacryloxyethyltrimethyl ammonium chloride
VTES	Triethoxysilane
VTMO	Trimethoxysilane
VMDS	Dimethoxymethylvinylsilane
ADE	Polymer of AM, DMC and VTES
ADM	Polymer of AM, DMC and VTMO
ADD	Polymer of AM, DMC and VMDS
ABIN	2,2′-Azobis (2-methylpropionitrile)
AR	Analytical reagent
