# Supplementary material for: Mechanisms of Covalent Bonds in Enhancing the Adsorption Stability of Clay–Polymer Gels in High-Temperature Environments
Source: Gels. 2025 Aug 9;11(8):623. doi: 10.3390/gels11080623 (PMC12385219; doi:10.3390/gels11080623)
Supplement: Supplementary file 1 [file gels-11-00623-s001.zip › gels-3794233-supplementary.pdf]

Supplementary Materials

Supplementary Tables:

Table S1: Rheological filtrate loss data of different 1% polymers added to a 4% base slurry at 25℃.

Type	600r	300r	AV(mPa·s)	PV(mPa·s)	YP(Pa)	FL (mL)
4% base slurry	16	10	8	6	2	19
4% base slurry/1% ADE	34	23	17	11	6	28.7
4% base slurry/1% ADM	27	17	13.5	10	3.5	25
4% base slurry/1% ADD	25	15	12.5	10	2.5	25.3

Table S2: Rheological filtrate loss data of different 1% polymers added to a 4% base slurry at 120℃.

Type	600r	300r	AV(mPa·s)	PV(mPa·s)	YP(Pa)	FL (mL)
4% base slurry	9.5	5	4.75	4.5	0.25	20.7
4% base slurry/1% ADE	16	9	8	7	1	11.7
4% base slurry/1% ADM	14	7.5	7	6.5	0.5	15
4% base slurry/1% ADD	14	7.5	7	6.5	0.5	14.3

Table S3: Rheological filtrate loss data of different 1% polymers added to a 4% base slurry at 150℃.

Type	600r	300r	AV(mPa·s)	PV(mPa·s)	YP(Pa)	FL (mL)
4% base slurry	11.5	6	5.75	5.5	0.25	20
4% base slurry/1% ADE	17	9	8.5	8	0.5	10.2
4% base slurry/1% ADM	14	7.5	7	6.5	0.5	8.7
4% base slurry/1% ADD	13	7	6.5	6	0.5	11

Table S4: Rheological filtrate loss data of different 1% polymers added to a 4% base slurry at 180℃.

Type	600r	300r	AV(mPa·s)	PV(mPa·s)	YP(Pa)	FL (mL)
4% base slurry	13	7	6.5	6	0.5	25
4% base slurry/1% ADE	10.5	5.5	5.25	5	0.25	15.3
4% base slurry/1%	10	5.5	5	4.5	0.5	15.3

ADM						
4% base slurry/1% ADD	11.5	6	5.75	5.5	0.25	14

Table S5: Rheological filtrate loss data of different 1% polymers added to a 4% base slurry at 210℃.

Type	600r	300r	AV(mPa·s)	PV(mPa·s)	YP(Pa)	FL (mL)
4% base slurry	11	6	5.5	5	0.5	30
4% base slurry/1% ADE	11.5	6	5.75	5.5	0.25	16
4% base slurry/1% ADM	11	6	5.5	5	0.5	17
4% base slurry/1% ADD	6	4	3	2	1	15

Table S6: The data results of three organosilicon polymer inhibitors and commonly used inhibitors subjected to 16-hour rolling aging on shale cuttings at 120℃, with measurements taken three times and averaged.

Type	120℃ Recovery (%)		
Deionized	35.5	33.35	30.85
1% ADM	87.75	89.85	88.2
1% ADE	88.2	87.2	86.36
1% ADD	85.3	88.05	89.65
5% KCl	54.1	56.4	59.62
1% Polyamine	76.05	80.6	78.32

Table S7: The data results of three organosilicon polymer inhibitors and commonly used inhibitors subjected to 16-hour rolling aging on shale cuttings at 150℃, with measurements taken three times and averaged.

Type	150℃ Recovery (%)		
Deionized	24.5	26.78	25.32
1% ADM	85.2	87.2	84.32
1% ADE	82.4	82.25	85.59
1% ADD	82.2	81.55	83.9
5% KCl	40.4	44.39	39.82
1% Polyamine	61.6	60.25	59.3

Table S8: The data results of three organosilicon polymer inhibitors and commonly used inhibitors subjected to 16-hour rolling aging on shale cuttings at 180℃, with measurements taken three times and averaged.

Type	180℃ Recovery (%)		
Deionized	16.05	18.3	17.96
1% ADM	83.35	80.3	81.32
1% ADE	82.45	86.7	83.7

1% ADD	88.2	87.32	91.42
5% KCl	32.14	30.1	28.3
1% Polyamine	63.6	60.69	58.36

Table S9: The data results of three organosilicon polymer inhibitors and commonly used inhibitors subjected to 16-hour rolling aging on shale cuttings at 210℃, with measurements taken three times and averaged.

Type	210℃ Recovery (%)		
Deionized	12.3	10.65	13.33
1% ADM	80.7	79.32	84.32
1% ADE	89.9	86.52	81.94
1% ADD	91.25	89.6	90.5
5% KCl	20.2	18.8	21.35
1% Polyamine	50.9	53.48	51.69

Table S10: The content of other elements on the surface of shale after rolling aging at 180℃ for 16 hours.

Element	wt%
C	13.95
N	0.00
O	35.84
Mg	0.90
Al	10.69
Si	27.60
K	5.06
Ca	0.31
Fe	5.65

Table S11: The content of other elements on the surface of shale after being treated with a 1% ADE polymer solution at 180°C for 16 hours of rolling aging.

Element	wt%
C	42.22
N	0.00
O	28.19
Mg	0.99
Al	5.01
Si	11.94
K	2.13
Ca	7.83
Fe	1.68

Table S12: The content of other elements on the surface of shale after being treated with a 1% ADM polymer solution at 180°C for 16 hours of rolling aging.

Element	wt%

C	37.61
N	0.86
O	28.90
Mg	0.68
Al	7.07
Si	16.39
K	2.88
Ca	1.59
Fe	4.00

Table S13: The content of other elements on the surface of shale after being treated with a 1% ADD polymer solution at 180°C for 16 hours of rolling aging.

Element	wt%
C	15.00
N	0.00
O	38.79
Mg	0.91
Al	9.95
Si	25.20
K	4.01
Ca	0.36
Fe	5.78

Supplementary Figure:

Scanning Electron Microscope (SEM) and Energy Dispersive X-ray Spectroscopy (EDS) Supplementary Data:

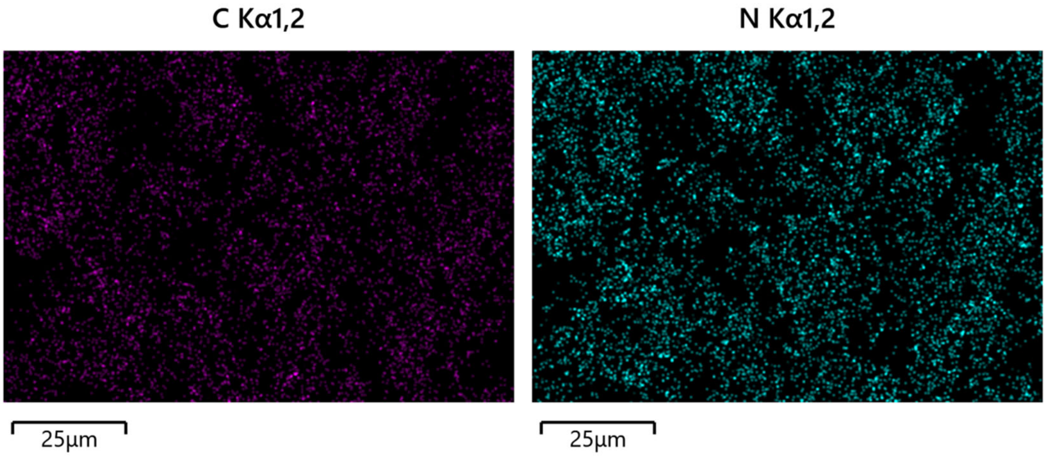

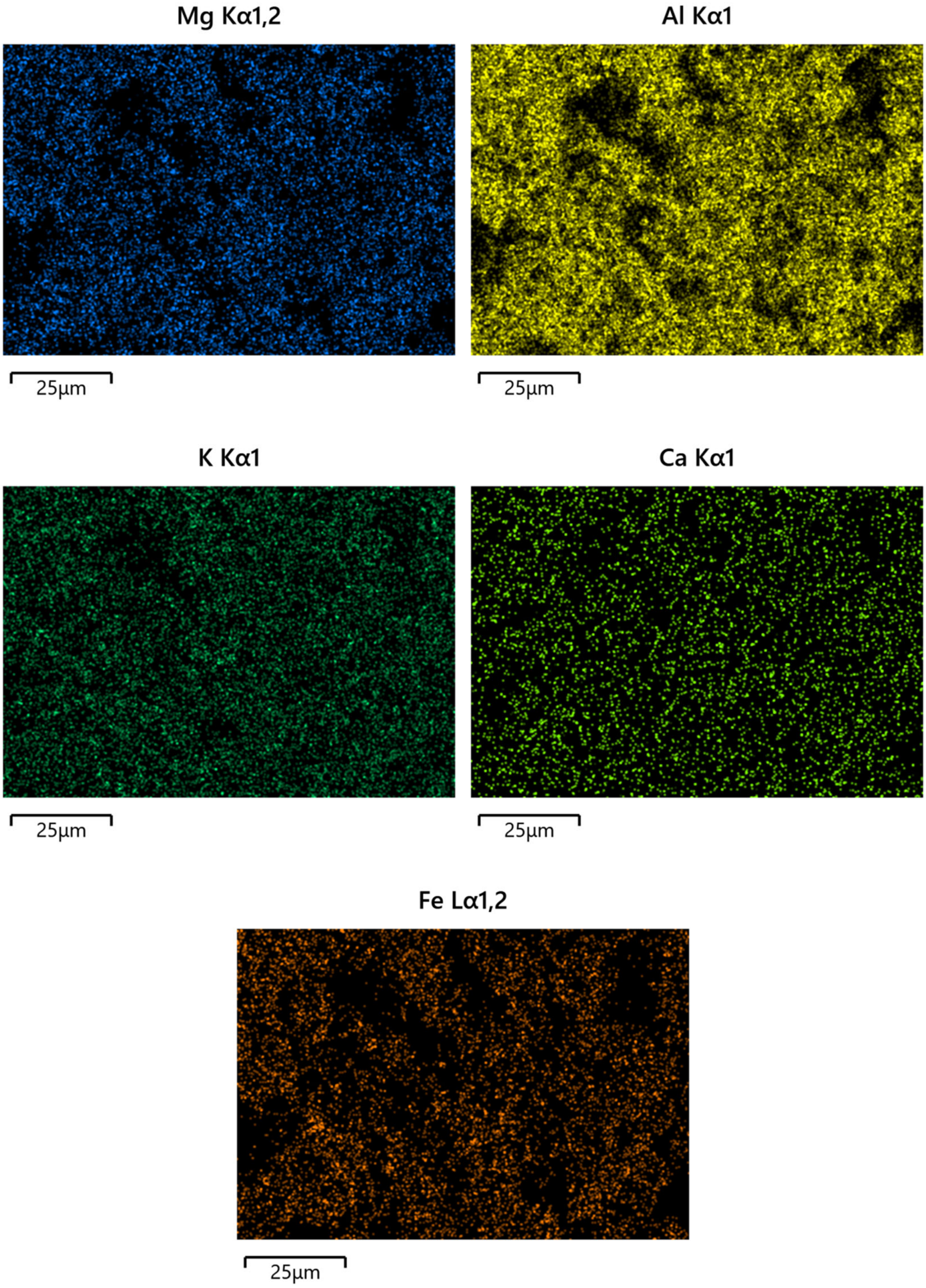

Figure S1: Distribution of other elements on the shale surface after rolling aging for 16 h at 180°C.

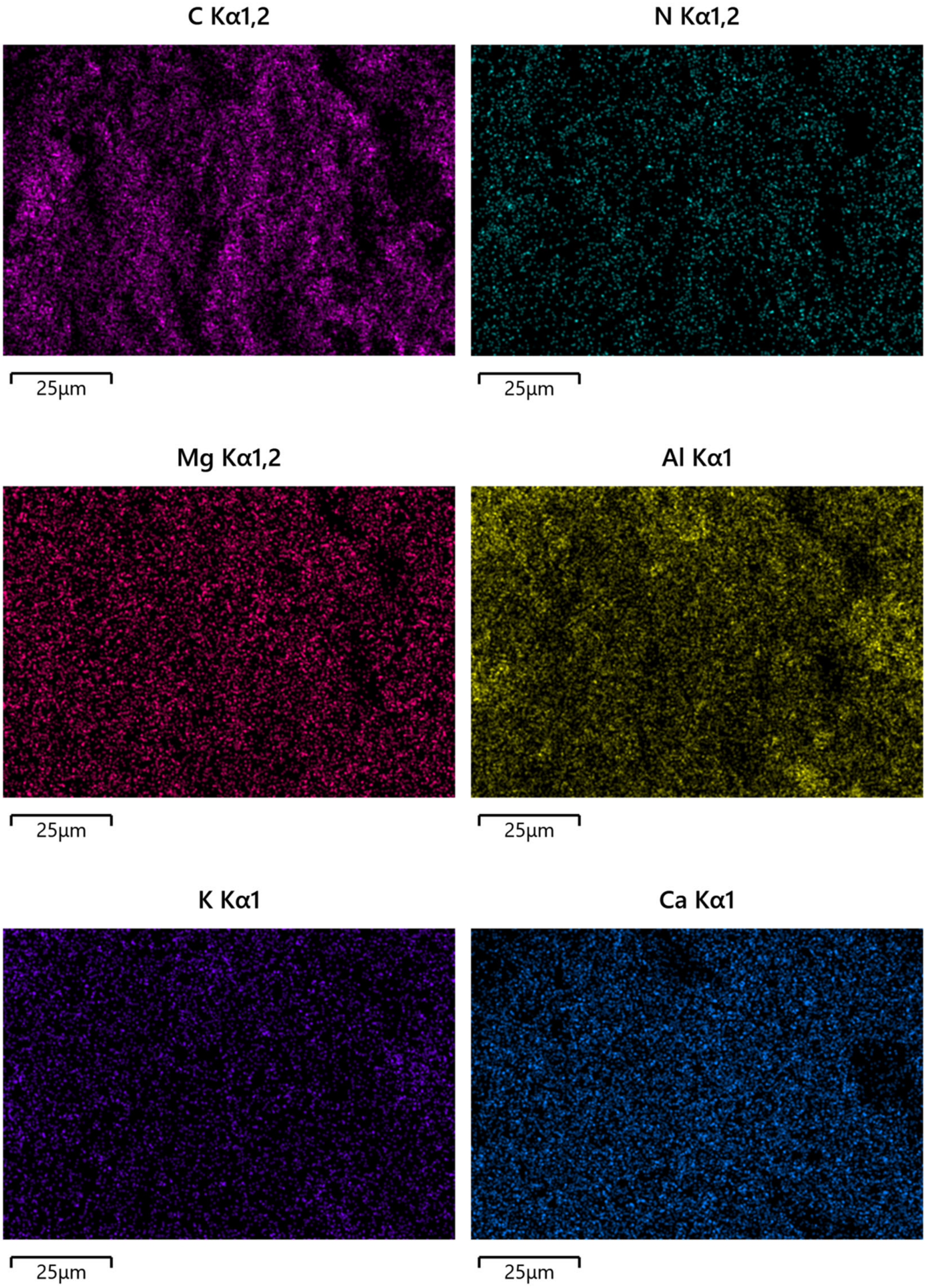
C Kα1,2
N Kα1,2
25μm
25μm
Mg Kα1,2
Al Kα1
25μm
25μm
K Kα1
Ca Kα1
25μm
25μm

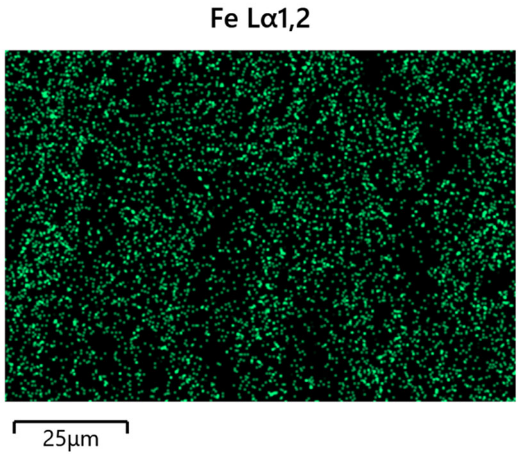

Figure S2: Distribution of other elements on the shale surface modified by 1% ADE polymer solution after rolling aging at 180°C for 16 h.

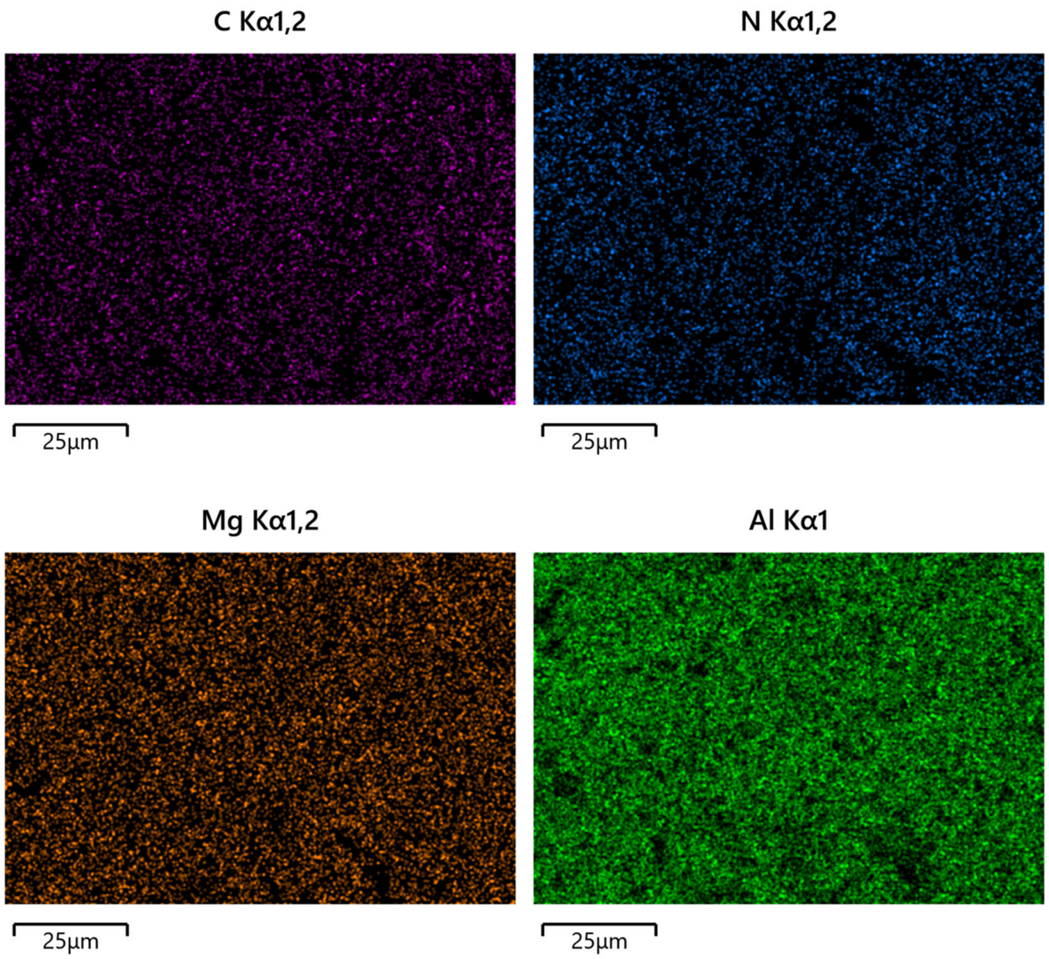

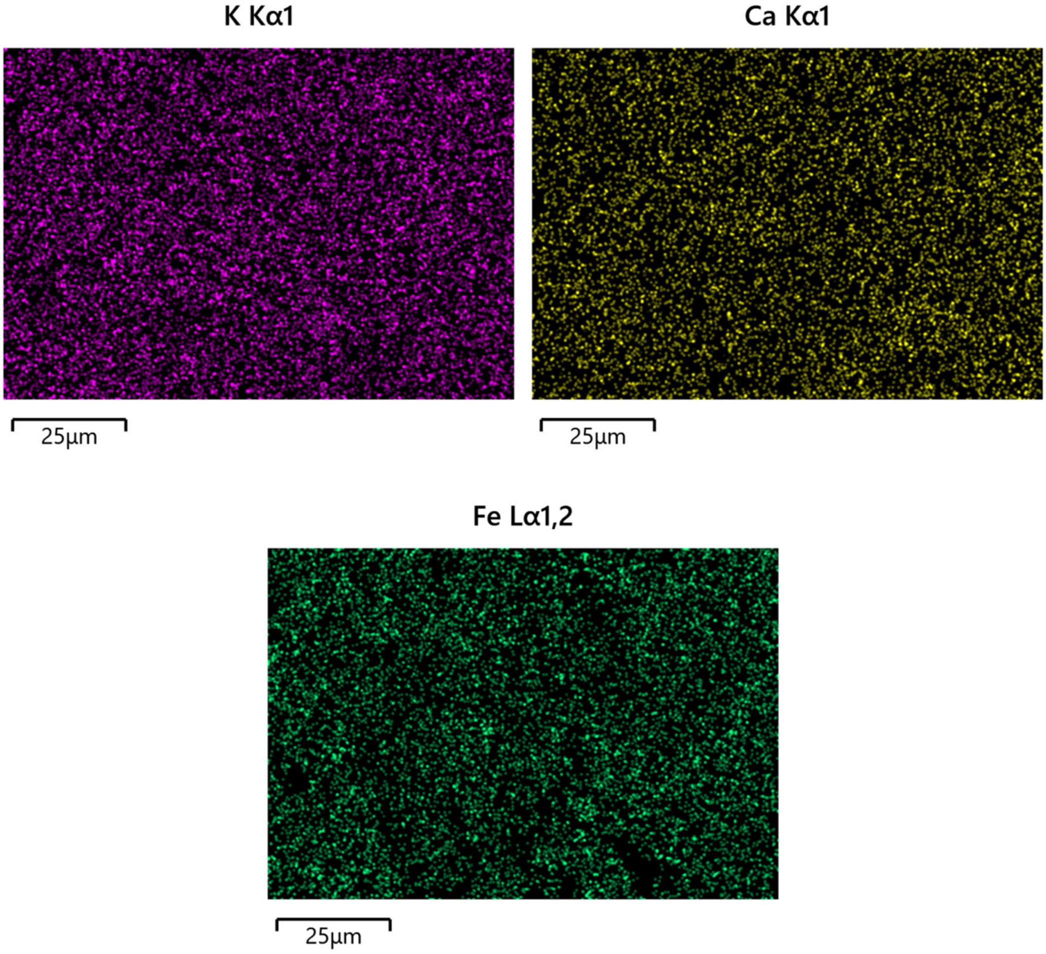

Figure S3: Distribution of other elements on the shale surface modified by 1% ADM polymer solution after rolling aging at 180°C for 16 h.

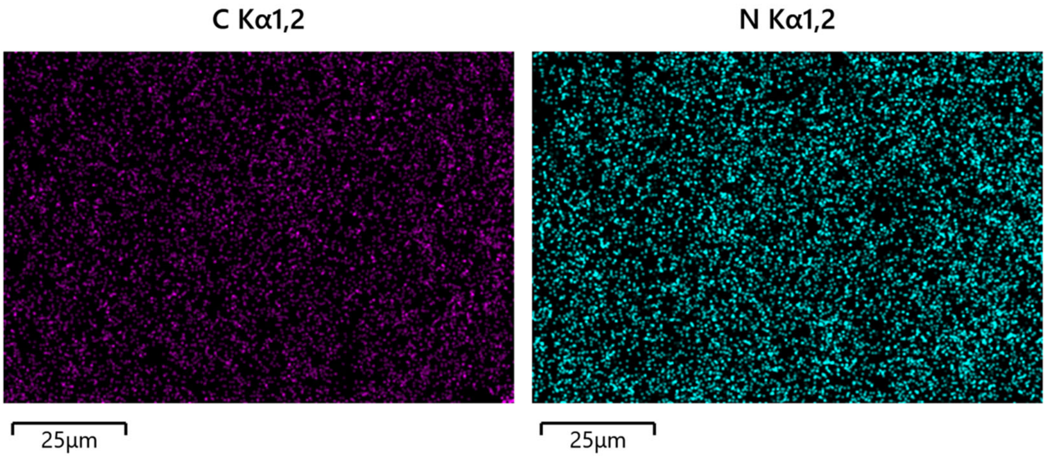

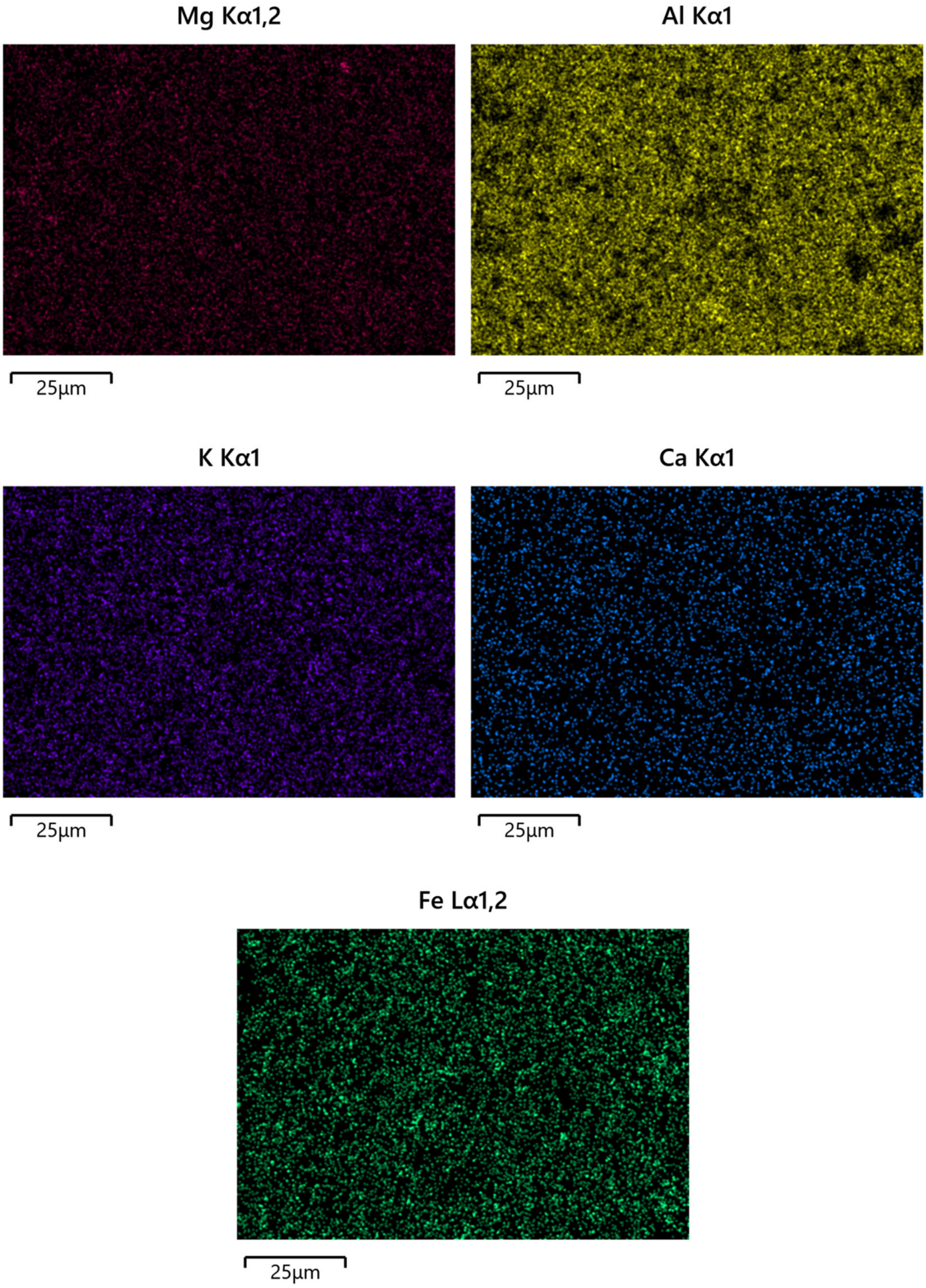

Figure S4: Distribution of other elements on the shale surface modified by 1% ADD polymer solution after rolling aging at 180°C for 16 h.
